# Image-Based Learning to Measure the Space Mean Speed on a Stretch of Road without the Need to Tag Images with Labels

**DOI:** 10.3390/s19051227

**Published:** 2019-03-11

**Authors:** Jincheol Lee, Seungbin Roh, Johyun Shin, Keemin Sohn

**Affiliations:** Department of Urban Engineering, Chung-Ang University, 84 Heukseok-ro, Dongjak-gu, Seoul 156-756, Korea; royalcity01@cau.ac.kr (J.L.); sbr444@cau.ac.kr (S.R.); olfy1021@cau.ac.kr (J.S.)

**Keywords:** space mean speed, convolutional neural network (CNN), cycle-consistent adversarial network (CycleGAN), traffic surveillance, traffic prediction

## Abstract

Space mean speed cannot be directly measured in the field, although it is a basic parameter that is used to evaluate traffic conditions. An end-to-end convolutional neural network (CNN) was adopted to measure the space mean speed based solely on two consecutive road images. However, tagging images with labels (=true space mean speeds) by manually positioning and tracking every vehicle on road images is a formidable task. The present study was focused on naïve animation images provided by a traffic simulator, because these contain perfect information concerning vehicle movement to attain labels. The animation images, however, seem far-removed from actual photos taken in the field. A cycle-consistent adversarial network (CycleGAN) bridged the reality gap by mapping the animation images into seemingly realistic images that could not be distinguished from real photos. A CNN model trained on the synthesized images was tested on real photos that had been manually labeled. The test performance was comparable to those of state-of-the-art motion-capture technologies. The proposed method showed that deep-learning models to measure the space mean speed could be trained without the need for time-consuming manual annotation.

## 1. Introduction

**Figure 1:**
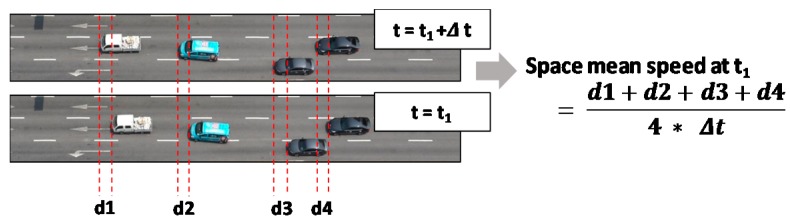
Conceptual explanation of space mean speed.

It is difficult to use existing traffic surveillance systems to directly measure the traffic density and space mean speed. Chung et al. [1] introduced a convolutional neural network (CNN) that can be used to measure traffic density based solely on a road image. This shows that the space mean speed could be measured from two consecutive photos taken over a short time interval. The contribution of the present study is two-fold: a robust CNN to measure the space mean speed on a road stretch was developed based solely on traffic images, and an innovative way was devised to circumvent difficulties in tagging images with true space mean speeds (=labels). For a given stretch of road the most definite method to measure the space mean speed for a given moment is to average the instantaneous speeds of all the vehicles (see Figure 1). If this measurement is possible, a profile of the space mean speeds along a time axis could be obtained, which is the most accurate indicator of the traffic dynamics of a road segment. However, there currently is no direct method to observe such a speed profile based on spot detectors that prevail in traffic surveillance.

It is conceptually easy to compute the space mean speed using two consecutive aerial photos of a road stretch taken over a short time interval (see Figure 1). Once the locational shift of the vehicles is measured and averaged by the vehicle count, the true space mean speed can be obtained. In principle, the space mean speed can be computed easily once each vehicle’s motion is captured.

In computer vision studies, many engineering-based methodologies were developed for motion capture within images. The background subtraction method has widely been adopted to detect and track vehicles in video images. Despite the successful measurement of vehicle movement, the method depends largely on engineering judgement to determine many threshold values in advance [2,3,4]. An optical flow method has also been widely adopted for motion capture, which derives motion vectors of moving vehicles from video shoots. This method was also successful in measuring the speed of vehicles on a road stretch [5,6,7]. However, these two methods are far from a data-driven approach. In other words, the measurement performance cannot be improved by providing more image data.

Besides such engineering methodologies, deep-learning models have recently been adopted to detect and track objects in video images [8,9,10]. However, deep neural networks require a large labeled dataset with bounding boxes for training and testing tasks. Drawing a bounding box for each vehicle in every training image would be a formidable task.

A summary of previous research shows that the former two methods depend largely on engineering judgement despite the higher level of accuracy, whereas the latter deep-learning model required considerable effort to tag images with labels including bounding boxes. The present study developed an end-to-end deep-learning model to collectively measure the space mean speed on a road stretch from two consecutive images with neither the need of engineering judgement nor the need to draw bounding boxes.

Our previous study [1] proved that a lighter CNN is sufficient to collectively count vehicles within an image without detecting and tracing individual vehicles. The CNN model was trained with only the vehicle count as a label. The present study adapted this simple scheme to measure the space mean speed in a road stretch based solely on two consecutive road images without detecting and tracing individual vehicles. However, this end-to-end model still requires much effort to label images with true space mean speeds. Such a task of labeling is a bottleneck since vehicles moving distances between two consecutive images should be measured manually (see Figure 1). The present study presents an innovative way to circumvent this labor-intensive labeling task.

For supervised learning, facilitating the data acquisition process is as important as establishing the model architecture. It is well known that machine-learning performance depends more on the amount of training data than on what kinds of models are adopted [11]. In the present study, an approach to securing a large amount of labeled data without the need for labor-intensive human effort was devised. An actual road condition is mimicked by a reliable traffic simulator, and then animation images generated via the traffic simulator are used to train models. This approach offers great flexibility by securing an unlimited amount of pre-labeled data for measurement of the space mean speed. This scheme allows an analyst to produce labeled images for various traffic conditions. However, the so-called reality gap problem remains as to whether a CNN trained on cartoon-like naive images that are provided by a simulator can properly work for real photos [12,13].

To tackle the problem, a state-of-the-art technology developed in the computer vision field was adopted. The trend of deep learning is being switched from supervised learning to unsupervised learning, due to the difficulty in securing labeled data for training. Goodfellow et al. [14] scored a breakthrough by devising the generative adversarial network (GAN). A GAN synthesizes the imaginary images of a specific domain from an arbitrary probabilistic distribution, after being trained on images that belong to the domain. As a result, the synthesized images are difficult to distinguish from real images. More recently, based on the concept of GAN, Zhu et al. [15] invented a cycle-consistent adversarial network (CycleGAN) that transforms images between two different domains. A CycleGAN trained on unpaired images can map an image that belongs to one domain into its corresponding image that belongs in another domain, with the context of the original image unchanged. The CycleGAN represents a significant leap in generating images by eliminating the need to collect paired or labeled image data. The present study adopted a CycleGAN to synthesize a seemingly realistic traffic photo from a naive animation image generated by a traffic simulator.

The purpose of utilizing the CycleGAN in the present study was to automatically create labeled images to train the proposed CNN to measure the space mean speed. The synthesized photos are perfectly labeled, since they originate from simulation that can be controlled by an analyst. Hence, a CycleGAN creates a seemingly realistic image with an exact label for any traffic condition that a traffic simulator could mimic. However, this scheme depends on an assumption that there is a reliable traffic simulator that can mimic real traffic conditions. Fortunately, several reliable traffic simulators such as Vissim, Paramics, and Corsim are available and widely accepted in the field of transportation studies. Recently in robotics, this kind of domain randomization skill along with a reliable simulator is being widely adopted to overcome the difficulty in securing labeled data to train robots [12,13].

The next section introduces the exact definition of the space mean speed and its theoretical and practical importance in traffic engineering. The third section describes the architecture of the proposed CNN model to measure space mean speeds. In the fourth section, the principle of how a CycleGAN synthesizes images that mimic a specific domain is described. Details of how to acquire image data to train and test the proposed models is accounted for in the fifth section. The modeling framework and its solution algorithm are introduced in the sixth section. Training and testing results from measurements of the space mean speed are presented and compared with those from state-of-the-art computer vision algorithms in the seventh section. The last section draws conclusions and suggests further studies to expand the present study.

## 2. An Overview of Space Mean Speed

Space mean speed is rigorously defined as the average speed of vehicles running on a road segment of a certain length at a given instant, whereas time mean speed is defined as the average speed of vehicles crossing a cross-section of road during a certain period. It should be noted that the space mean speed is more important for both the theoretical and practical aspects in the field of transportation engineering. Theoretically, the basic relationship (Equation (1)) among traffic flow, density, and speed holds only when the space mean speed is engaged. The space mean speed is thus inevitable for developing traffic flow theory, and it is critical for modeling the exact behavior of traffic flows. Practically, the time required to traverse a road segment is basic information that travelers use to plan a journey. The average travel time is accurate when computed by dividing the length of a road segment by the space mean speed. Thus, the space mean speed is the basis on which traffic information for navigation services is generated.

(1) $$q=k\times \bar{v}$$

where q= flow rate, k= density, and $\bar{v}=$ the space mean speed.

The space mean speed measured continuously over time makes it possible to recognize an immediate change in traffic conditions. Moreover, according to Hall [16], a distribution of space mean speeds is identical to the true distribution of speeds, whereas time mean speeds measured over time at a fixed point do not match the true distribution. However, an intrinsic problem lies in the fact that the space mean speed cannot be measured in the field using conventional surveillance systems based on spot detectors. If every vehicle on a given stretch of road is equipped with an on-board unit that transmits its speed on a short-term basis, the space mean speed could easily be measured. However, such a connected environment will not be available any time soon.

In the early stages of traffic engineering, Edie [17] introduced another concept for deriving representative speed. He averaged vehicle speeds across a time-space domain [see Equation (2)]. This scheme provides the rationale for using probe vehicles to obtain an average speed. To do so, times spent, and distances traveled by probe vehicles are collected in a time-space domain. However, the average speed cannot be a space mean speed under the rigorous definition given above, and the rate of market penetration has great influence on measuring the speed in the field.

(2) $${\bar{v}}^{\prime }=\frac{\sum_{i=1}^{n}x_{i}}{\sum_{i=1}^{n}t_{i}}$$

where $x_{i}$ = the distance traveled by the *i*-th vehicle in the time-space area and $t_{i}$ = the time spent by the *i*-th vehicle in the time-space area.

Due to the difficulty in measuring the space mean speed, many researchers have attempted to estimate it from the travel time over an extended distance by tracking vehicles based on their signature across a series of loop detectors [18,19,20,21], but there have been few practical implementations yet. Other researchers adopted mathematical ways to approximate the space mean speed from the time mean speed and its variance that can be easily measured using a conventional spot detector [22,23,24].

The present study introduces the use of cutting-edge technology to directly measure the space mean speed. A CNN circumvents the complications regarding the measurement of space mean speed owing to its capability of implicitly detecting and tracking objects based solely on images. An image-based prediction for traffic speeds is prevalent now in the field of transportation studies [25,26,27]. The next section introduces the architecture of a CNN established to measure the space mean speed.

## 3. A CNN Architecture that Measures the Space Mean Speed

It is plausible that a CNN could be trained to measure the space mean speed based solely on two consecutive images on a road stretch. A human easily recognizes vehicle movements when consecutive images are overlapped with a fixed background. The present study was initiated by the notion that a CNN could mimic this human ability in an end-to-end manner. A CNN is expected to identify the pixel-by-pixel differences between the two images and to recognize the changes in vehicle positions. It is a rational assumption that the space mean speed could be estimated by scaling these changes. Many researchers have devised various engineering tools to measure vehicle speeds based on this assumption [28,29,30]. However, these engineering-based models were more complex and less transferable than an end-to-end learning model.

An advanced deep CNN such as a the “You only look once” version 3 (YOLO v3) model can detect an individual object in video footage [31]. This technology could also be used to estimate the space mean speed. One drawback, however, is the need for an additional treatment for YOLO v3 to track an individual vehicle after detection. Tracking a vehicle by its estimated bounding box entails an intrinsic error whereby the bounding box cannot maintain its shape and size over time. Moreover, another drawback arises when crowded and small objects cannot be detected. The latter problem is more critical when the model is applied to aerial photos for a long road segment wherein an individual vehicle seems very small. A road segment may also encompass severely congested traffic conditions. The present study began with the expectation that a CNN could measure the space mean speed as a whole even for a road stretch under severely congested conditions without the step-by-step procedure of detecting and tracking.

It is clear that monitoring the service level of road traffic is less strict than detecting objects for an autonomous vehicle. Measuring the space mean speed for the purpose of traffic management and operation permits errors to a certain extent. A CNN is thus sufficient to collectively measure the space mean speed. The CNN model devised by Chung el al. [1] to measure traffic density was adapted for the present study to measure the space mean speed. The CNN required two images as input and produced one-dimensional output that represented the space mean speed. The architecture of the CNN was chosen after testing as many plausible model structures as possible. Even though this scheme seems naïve, it is also unknown how the architecture of advanced deep neural networks such as Imagenet, Alexnet, VGG-19, or Googlenet were chosen.

**Figure 2:**
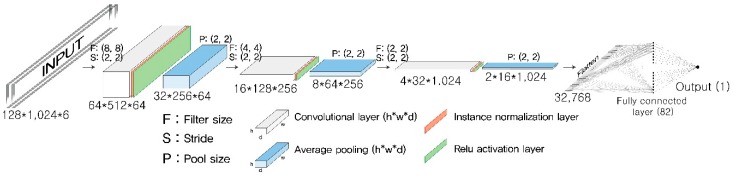
Architecture of the CNN used to measure the space mean speed.

The chosen architecture of the present CNN model is shown in Figure 2. The CNN architecture was built by stacking three convolutional blocks, each of which was composed of a convolutional layer, an instance normalization layer, a rectified linear unit (Relu) activation layer, and an average pooling layer. Normalizing input feature maps on a layer-by-layer basis enhances the performance of a deep neural network to abstract features from images. The instance normalization computes the mean and standard deviation across spatial dimensions independently of each feature map within a batch input [32], unlike the batch normalization that computes them across an entire batch of images [33]. A fully connected layer was attached to the end of the last convolutional block after flattening the last feature map. The output layer was set as a single real number representing a space mean speed, so that the role of the model could be the same as that of a general regression model.

## 4. Using CycleGAN to Synthesize Realistic Photos

Modern machine learning technology has advanced from a state of depending on supervised learning models that require a large amount of labeled data to a state of developing generative models. A generative model creates hypothesized output that resembles the ground truth after being trained on unlabeled data. The CycleGAN represents a breakthrough in translating images between two different contexts without paired images for training. Two independent sets of images, each of which corresponds to a specific domain, are used for training the model. For example, a CycleGAN could convert a zebra image into a horse image, and vice versa, after training the model using unpaired zebra and horse photos. This scheme can be directly applied to fulfilling the present objective to convert an animated image of traffic into a real traffic photo.

CycleGAN has a relatively simple structure that involves two different types of deep CNNs: a generator and a discriminator. The generator maps an image in one domain into a corresponding image in another domain. Two different mapping directions exist between two domains (X and Y), and two separate mapping functions are necessary (*G: X→Y* and *F: Y→X*). In fact, the two generators take the same form, but come to have different weight parameters after being trained.

The discriminator acts as a tester to identify whether an image is synthesized or true. Two discriminator functions are necessary ($D_{X}$ and $D_{Y}$) for two domains (X and Y), respectively. $D_{X}$ aims to recognize whether an image belongs to domain X, and $D_{Y}$ aims to recognize whether an image belongs to domain Y. The discriminator is a type of CNN classifier that retrieves the probability that an input image belongs to a given domain. The two discriminators have the same CNN structure, but they obtain different weight parameters after being trained.

The goal of a CycleGAN is to train the generator to create images that the discriminator could not distinguish as synthesized. The loss function was set up to fulfill this objective. First, the adversarial loss for both mapping functions was established, expressed by Equations (3) and (4), respectively, to be maximized during learning. The first term of the loss function represents the log-likelihood that a real image in a domain is proven to be real, whereas the second term stands for the log-likelihood that a synthesized image turns out to be synthesized. The loss function takes the average of individual log-likelihoods across a sample extracted from the true probabilistic density of each domain:

(3) $${ℒ}_{GAN1}\left(F,D_{X},X,Y\right)=E_{x\sim P_{data}\left(x\right)}\left[logD_{X}\left(x\right)\right]+E_{y\sim P_{data}\left(y\right)}\left[\log(1-D_{X}\left(F\left(y\right)\right)\right]$$

(4) $${ℒ}_{GAN2}\left(G,D_{Y},X,Y\right)=E_{y\sim P_{data}\left(y\right)}\left[logD_{Y}\left(y\right)\right]+E_{x\sim P_{data}\left(x\right)}\left[\log(1-D_{Y}\left(G\left(x\right)\right)\right]$$

The second loss secures the consistency between the original image x∈X (or *y* ∈Y) and the cycled image *F*(*G*(*x*)) [or *G*(F(y))] after going through double mappings. The pixel-by-pixel difference between the images is minimized so that the cycled image is as close to the original image as possible. This cycle consistency loss is separately set up for each domain as shown in Equation (5) and minimized during learning:

(5) $${ℒ}_{cycle}\left(G,F,X,Y\right)=E_{x\sim P_{data}\left(x\right)}\left[F\left(G\left(x\right)\right)-x_{1}\right]+E_{y\sim P_{data}\left(y\right)}\left[G\left(F\left(y\right)\right)-y_{1}\right]$$

**Figure 3:**
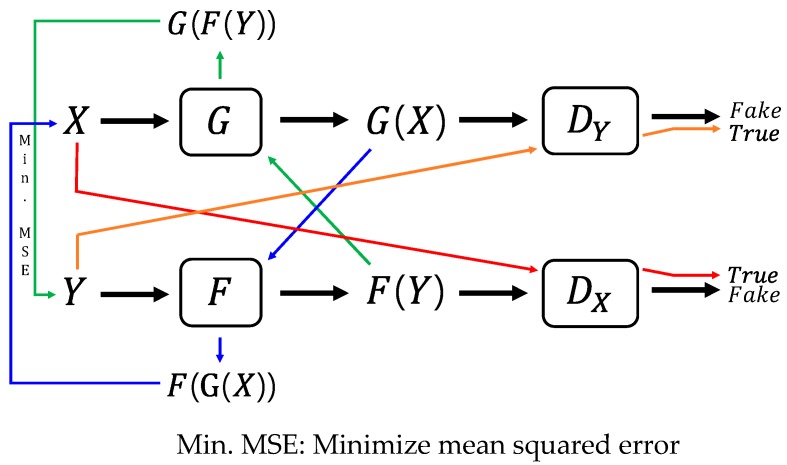
Optimization procedure for the CycleGAN.

The total loss function is the sum of the three loss functions above. A hyperparameter (λ) is adopted to balance the relative importance of loss functions. Equation (6) denotes the full loss function of a CycleGAN, in which the former two loss functions should be maximized with respect to $D_{X}$ and $D_{Y}$, and the last loss function should be minimized with respect to G and F. Equation (7) represents how to derive the two optimal generators (=mapping functions). This is a typical two-player minimax game that can be solved iteratively with generators and discriminators fixed alternately. The diagram for the optimization procedure is shown in Figure 3:

(6) $$ℒ\left(G,F,D_{X},D_{Y}\right)={ℒ}_{GAN1}\left(F,D_{X},X,Y\right)+{ℒ}_{GAN2}\left(G,D_{Y},X,Y\right)+\lambda {ℒ}_{cycle}\left(G,F,X,Y\right)$$

(7) $$G^{*},F^{*}=\arg\;min_{F,G}\;max_{D_{X},D_{Y}}\;ℒ\left(G,F,D_{X},D_{Y}\right)$$

**Figure 4:**
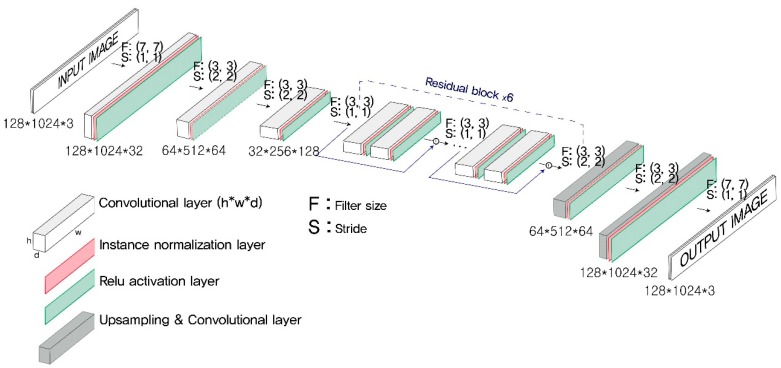
Architecture of the generator network.

The architecture of the generator was chosen after testing as many plausible alterations as possible, beginning with the model structure established originally by Johnson et al. [34]. The generator network was deeper than the discriminator network and included both convolutional and deconvolutional blocks (see Figure 4). The convolutional blocks extract features from an input image. The deconvolutional blocks attach to the end of the final convolutional block to rebuild an output image with the same dimensions as the input image, and preserve the features extracted by the former convolutional blocks. A convolutional block consists of three layers: a convolutional layer, an instance normalization layer, and a Relu activation layer.

Another advantage came from adopting residual blocks, as devised by He et al. [35]. The generator network had 6 residual blocks. Each residual block was composed of two convolutional blocks. Once a residual block receives an input tensor, it yields an intermediate output tensor by passing it through the subsequent layers. The final output tensor of the residual block is created by adding (or concatenating) the input tensor and the intermediate output tensor. The residual block helps prevent any loss of the original input features.

The deconvolutional block consists of an up-sample layer, a convolutional layer, an instance normalization layer, and a Relu layer. The deconvolutional block up-samples the previous feature map to rebuild an output image with the same dimensions as the input image.

**Figure 5:**
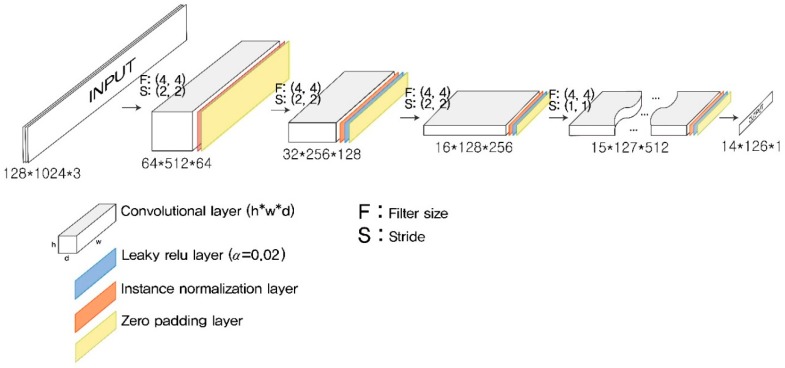
Architecture of the discriminator network.

The discriminator includes 4 convolutional blocks, each of which is composed of a convolutional layer, an instance normalization layer, and a leakyReLu activation layer. The leakyRelu converts negative input to a small positive value rather than suppressing the value to 0, which also considerably improves the performance of a deep neural network [36]. The discriminator showed better performance when a PatchGAN was adopted [37,38,39]. Accordingly, a two-dimensional tensor output (e.g., 14 × 126) was used for the discriminator rather than a single entropy output, so that each element of the tensor output could judge whether a part of the input image was true or synthesized. The ground-truth label had the same dimensions and was filled with binary values according to whether or not an input image was real. Figure 5 shows the chosen architecture of the discriminator networks.

## 5. Data Acquisition

**Figure 6:**
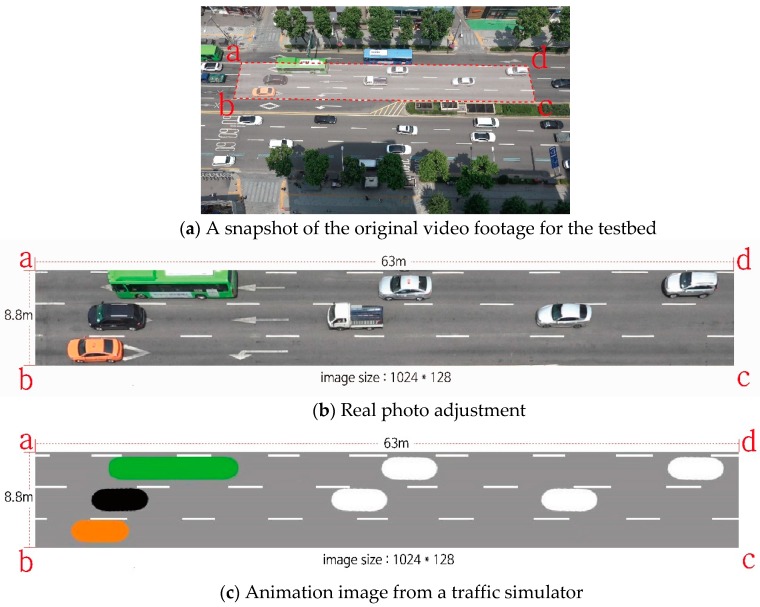
Real photo adjustment and animation image generation.

Two sets of image data were prepared to train CNNs for measuring the space mean speed and a CycleGAN for converting animation images into real photos. A stretch of road located in the south region of Seoul, Korea was selected as the testbed wherein a 4-h video was filmed. Raw video images were adjusted to correct distortions due to a camera lens. Since the raw images were taken by a high-end video camera with a long-distance view, the re-projection error was very small and all horizontal lane markers in each raw image were aligned. After calibration to mitigate camera distortions, three lanes corresponding only to through traffic were cropped with those of right and left turns excluded (see Figure 6a). A transformation was then applied to the cropped image to adjust orientation and viewpoint. The transformation required real-world coordinates for predefined reference points. The coordinates were directly measured in the field. The transformed image was resized to a resolution of 128 × 1024, as shown in Figure 6b. The OpenCV library provided all functions for the image transformations.

Such an original image with a bird-eye view is available in the Seoul metropolitan area owing to CCTVs mounted in every intersection. The CCTVs cover each intersection approach by changing direction and angle. Although the camera direction and angle vary, once a few reference points in the field are known, it is a trivial task to attain a necessary image with a rectangular shape as described above. We took bird’s-eye-view images on the testbed using our own video camera, since we had not attained permission to use CCTV images for the testbed.

Figure 6c shows a sample animation image generated from traffic simulation that mimics the real traffic of the testbed. It is very important to match the scales of the two images (Figure 6b,c). The adjusted photo (1024 × 128) covers a real road segment 63 m long and 8.8 m wide. The latter animation image was generated on the same scale, so that distances in the former image would be consistent with those in the latter image.

Space mean speeds were manually provided for 100 randomly chosen pairs of real photos. These images were used for the final test of the CNNs that had been trained on artificial images. It should be noted that there is no need to tag labels to real photos once the present test succeeds based on these 100 pairs of images. In order to train a CycleGAN two independent sets of images without labels were prepared: 500 randomly chosen animation images and 500 photos selected randomly from the video shoot.

The simulation environment was set identical to the real environment of the testbed. The real composition of vehicle types and the observed traffic volume were fed to the simulator. Traffic lights close to the left end of the testbed were applied to the simulated environment, so that the simulation could generate various traffic conditions that ranged from uncongested to stop-and-go. After a 30 min warm-up, animation images were collected for 4 h of simulation time. The time increment for the simulation was set sufficiently small (=0.1 s), so that the simulator could not move vehicles by more than the vehicle length. When a vehicle ran a distance longer than its length during a time interval between two consecutive images, it was difficult for the CNN to capture the distance as a movement.

**Figure 7:**
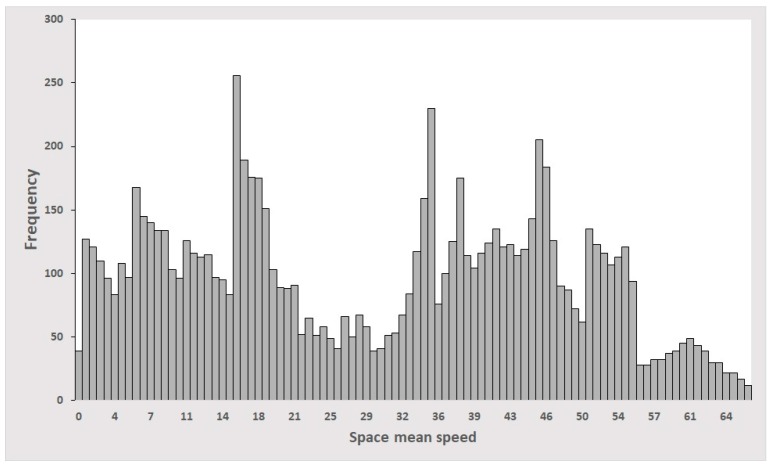
Distribution of space mean speeds for 9000 pairs of animation images.

The simulation period was set identical to the duration of the video (=4 h). Nine thousand pairs of images (=2 × 9000 images) were selected randomly from the simulated animation. Figure 6c shows a typical example of an animation image. A resolution of 128 × 1024 was commonly applied to both the real and animation images. These animation images had exact labels of space mean speeds extracted from the simulator. Figure 7 shows the distribution of space mean speeds for the chosen 9000 pairs of consecutive images. The space mean speed ranged from 0 to 67 KPH, which was consistent with the real traffic conditions in the testbed.

In summary, the data for training and testing models were obtained via a two-step procedure. The first step was to shoot video in the testbed, and the second step was to collect data from a simulation experiment that had been set identical to the testbed. This procedure was free from the burden of manually tagging labels to images. Although a single road segment was tested in the present study, the proposed data acquisition approach could be directly applied to any other road segments.

## 6. Modeling Framework

### 6.1. Training and Testing Models

**Figure 8:**
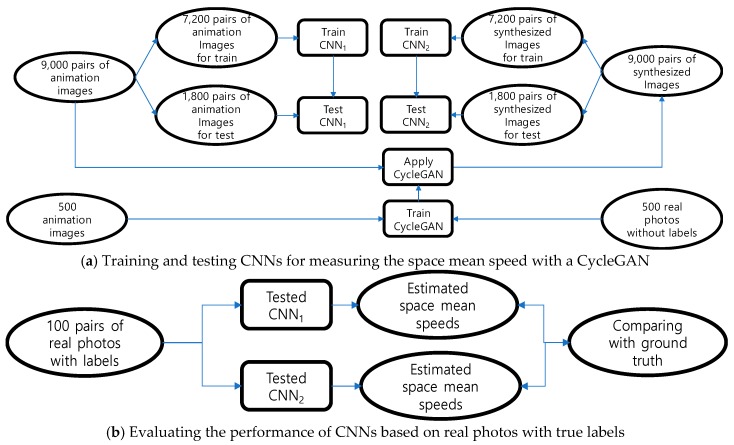
Training, testing, and evaluating the procedures of models.

The first CNN model (=CNN_1_) for measuring the space mean speed was trained on 9000 pairs of cartoon-like naïve animation images with exact labels. A CycleGAN was trained using two unpaired datasets (=a set of 500 real photos and a set of 500 animation images), as shown in Figure 8a. The trained CycleGAN generated 9000 pairs of synthesized images from the 9000 pairs of animation images, which were then used to train and test the second CNN (=CNN_2_) to measure the space mean speed. After training and testing the two CNNs for measuring the space mean speed, 100 pairs of real photos with true labels were used to finally evaluate their performances (see Figure 8b).

The goal of the present study was to ensure that both the CNNs that were trained and tested with artificial images could measure the space mean speed for a real road segment within a tolerable level of errors. CNN_2_ that was trained and tested on synthesized images was expected to outperform CNN_1_ depending on the naïve animation images, since the images synthesized by CycleGAN more closely approximated the real photos.

Each image with a resolution of 128 × 1024 was used as-is in order to train a CycleGAN, even though the original CycleGAN was developed with input resolutions of either 128 × 128 or 256 × 256. For the two CNNs to measure the space mean speed, 80% of the data were used for training, and the remaining 20% were reserved for testing.

### 6.2. Solution Algorithms and the Computation Environment

The basic loss of the two CNNs for measuring the space mean speed was identical to a general regression model. The discrepancy between the observed and the estimated space mean speeds was minimized. A stochastic gradient descent (SGD) algorithm was used to train the two CNNs for measuring the space mean speed. The SGD has been successfully applied to training deep learning models based on large-scale data. The algorithm saved considerable computation time, since it did not sweep the full batch of the dataset to compute a gradient of the loss function for an iteration. Instead, SGD updates the incumbent solution based only on a mini-batch, which is a small sample that is randomly selected from the entire training dataset without replacement. This sampling and updating procedure repeats until the training dataset is empty, which is referred to as an epoch. It is known that implementing multiple epochs ensures convergence to a local optimum. The recent successes gained with deep-learning models are among the results of this revolutionary algorithm. For details of the algorithm, readers are referred to Bottou [40].

**Figure 9:**
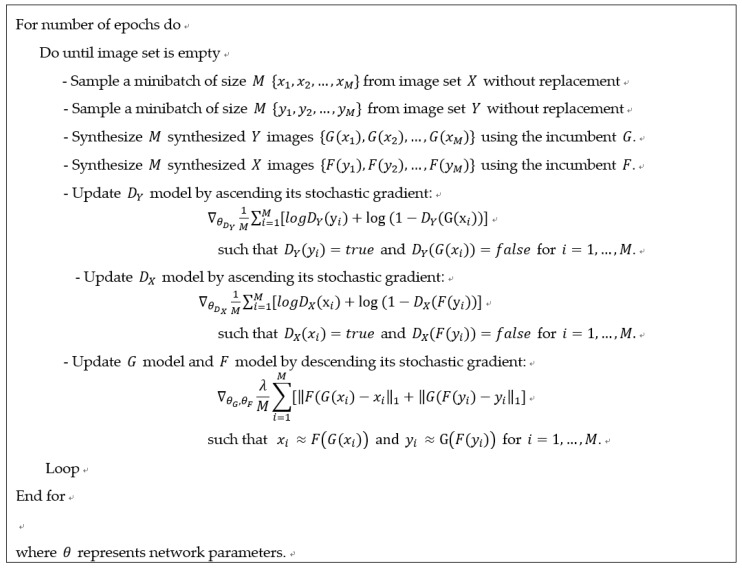
Solution algorithm for CycleGAN.

A SGD algorithm was also used to train a CycleGAN within the modeling framework suggested in Figure 8a. Generators and discriminators were updated alternately with the counterparts fixed at the incumbent models (see Figure 9). That is, images created by incumbent generators were used to update the discriminators, and then the generators were updated such that cycled images would closely approximate the original images. The mini-batch size M in the algorithm is a hyperparameter that should be chosen according to the computing capability. The hyperparameter was set as 3 within our computation environment.

The GPU utilized in the present study was a NVDIA Quadro M6000. The maximum number of epochs needed to reach convergence was set at 100, so that the total computing time would be within a practical range (=about 12 h). Several software libraries were mobilized to train a deep neural network on a GPU. Keras is a library that provides high-level deep-learning functions. Keras should be implemented with TensorFlow running at the back end to ensure that it can utilize TensorFlow’s low-level functions on a GPU. A Numpy library is also needed to handle array data with Python, a main programming language. These open-source libraries were downloaded from the Internet.

## 7. Model Results

### 7.1. Results from Mapping Images Using a CycleGAN

**Figure 10:**
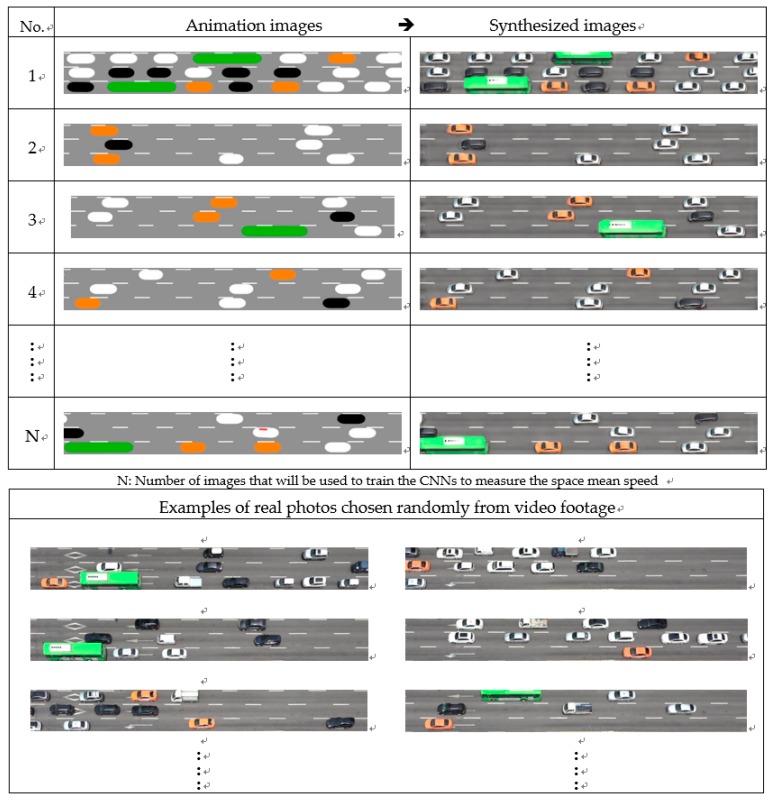
Comparison among animated, synthesized, and real images.

The trained CycleGAN was used to synthesize seemingly realistic photos from naïve animation images. Unfortunately, there is no robust measure to evaluate how similar a synthesized image is to a real photo. Authors of the original paper devised a performance index that was estimated using subjective evaluations from a group of people [15]. Such a human group evaluation was not used in the present study. Instead, Figure 10 shows several synthesized images with corresponding input images for intuitive judgement. For better comparisons, several real photos of the testbed were presented together, although they were not paired with the synthesized and animation images.

Intuitively, the synthesized images do not seem exactly the same as real photos, but it is apparent that they are much more realistic than naïve animation images. The similarity between the synthesized and real photos is a key to providing advantages in measuring the space mean speed in the field, which will be shown in the next sub-section.

**Figure 11:**
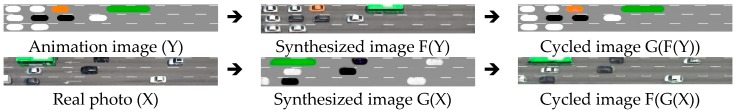
Mapping examples of CycleGAN.

It is also possible for a trained CycleGAN to map real photos into animation images, but results of this conversion were not introduced since they have no utility in traffic surveillance. Figure 11 indicates examples to show how similar the cycled images are to the originals. Because one of the loss functions to be minimized was set as the discrepancy between the original and cycled images, it was not surprising that images after double mapping are very similar to the original input images.

### 7.2. Results from Measuring the Space Mean Speed Using CNNs

**Figure 12:**
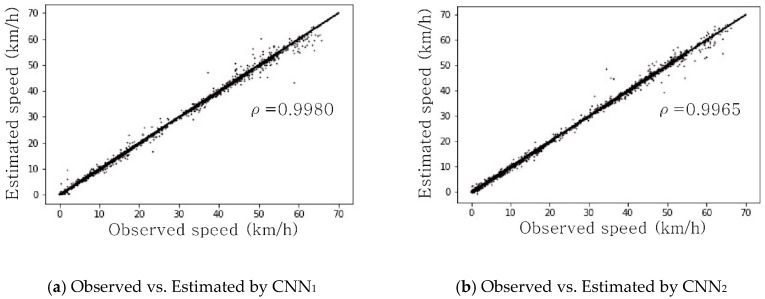
Comparison of observed and estimated space mean speeds estimated using artificial images. ρ: Correlation coefficient.

Measuring the space mean speed from artificial images were very successful in terms of three different performance measures, each of which accounts for the discrepancy between estimated speeds and ground-truth speeds elicited from simulators. Table 1 shows the evaluation results based on the three performance indices, and more intuitive comparisons are shown in Figure 12, which depicts the XY-plots between observed and estimated space mean speeds. The plots were drawn under the assumption that space mean speeds elicited from the simulation were the observed space mean speeds.

Even though both models showed good simulation performance, effectiveness in the field is a separate issue because the models were trained and tested only on artificial images that originated from a simulation. To finally assess the utility of the proposed approach in the field, the space mean speed was manually measured for 100 pairs of real photos for the testbed. It should be noted that there would be no need for such a manual labeling task once the proposed approach passes the final evaluation.

**Figure 13:**
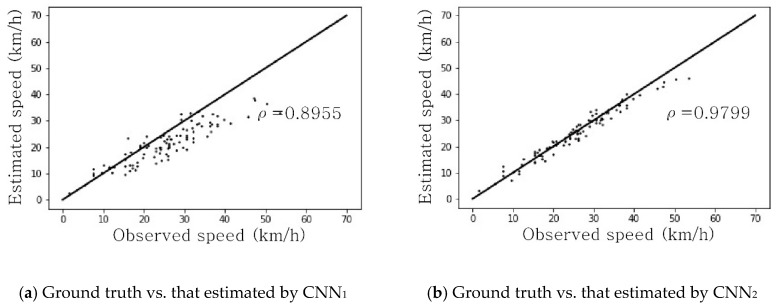
Comparison between observed and estimated space mean speeds estimated using real photos. ρ: Correlation coefficient.

For the 100 pairs of real photos with perfect labels, the two trained CNNs predicted the space mean speed. The predicted speeds are compared with the ground-truth speeds in Table 2. XY-plots between the ground truth and the estimated space mean speeds are shown Figure 13. As expected, the CNN_2_ trained on the images synthesized by CycleGAN performed better in terms of all three indices than the CNN_1_ trained on the animation images from a traffic simulator. The more meaningful results were that the performance measures had not deteriorated much by comparison with those from synthesized images. This implies that a CycleGAN has sufficient potential to overcome the reality gap and thus the space mean speed can be measured for any road segment without the burden of labeling, once a reliable traffic simulator is available. If a more robust CycleGAN is developed in the future, the measurement accuracy could be enhanced considerably.

**Figure 14:**
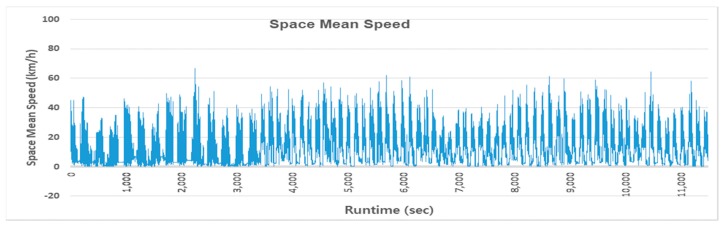
Profile of the space mean speed for the testbed.

Using the CNN_2_ trained on synthesized images, a profile of the space mean speed was accomplished using the 4-h real video footage for the testbed (see Figure 14). The space mean speed was measured every 0.1 s from the video footage. To the best of our knowledge, such a speed profile had never been measured in real time. This continuous speed profile contains more information on traffic conditions than any other forms of traffic surveillance system could provide.

### 7.3. Performance Comparisons with Other Methodologies

**Figure 15:**
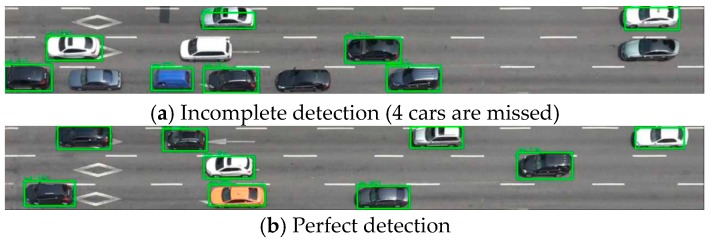
A success and a failure in YOLO detection.

Three state-of-the-art computer vision technologies were mobilized to confirm that the performance of the proposed methodology is competitive. Besides these technologies, a CNN model trained on images drawn by a 3-D car model was also compared with the proposed methodology. The reference models were tested against the same dataset (=100 pairs of images with true labels) as used in the final validation. First, YOLO v3 was employed to measure the space mean speed for the dataset. Figure 15 shows a successful and a failed YOLO. The first image shows the YOLO is not error-free when the density of objects gets larger, as mentioned in the original paper [31].

After detecting individual cars, the car speed was computed by tracing the bounding boxes. In this manner, the space mean speed was computed for the testbed. While tracing the bounding boxes, there was another error because a YOLO cannot maintain a constant bounding box. It was not accurate to trace the coordinates of the upper left corner of the variable bounding box. Also, the error was exacerbated since the space mean speed was computed only for vehicles detected. The proposed approach using a Cycle GAN also entails a measurement error. However, the detection error in YOLO more seriously influenced the measurement of the space mean speed.

**Figure 16:**
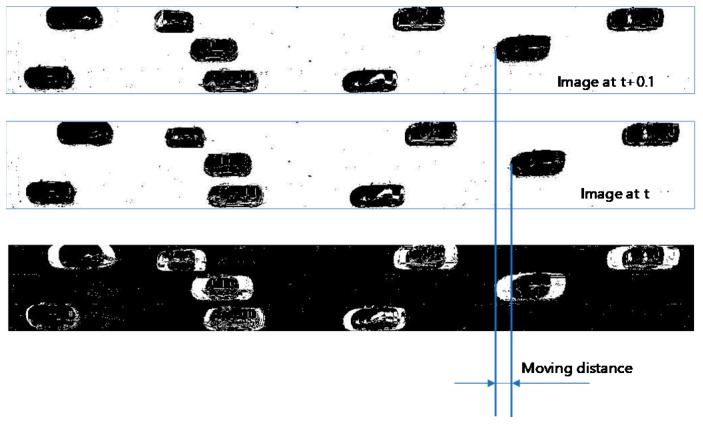
A description of the background subtraction methodology.

The second reference methodology was a background subtraction. Figure 16 shows how to measure moving distances of vehicles based on a background subtraction method. A background image and two pairs of photos taken in 0.1 s. intervals were prepared. The background image was obtained such that each cell of the image took the median value of the same cell values of all 4-h video shoots. The top two images in Figure 16 were obtained from the difference in cell values between two consecutive images. The threshold value to judge whether the difference originates from a vehicle’s presence was set as 17. A cell with a difference larger than this threshold value was assigned 0; otherwise, it was assigned 255. The bottom image was created by subtracting the top image from the middle image and reversing the subtracted image. To measure the moving distances of vehicles, white blobs were recognized and then their average horizontal lengths were measured and summed. The sum of the average lengths was then divided by 2, since each vehicle had two white blobs. The space mean speed was computed by dividing the sum of the distances by the vehicle count. Detecting white blobs also required engineering judgement.

**Figure 17:**
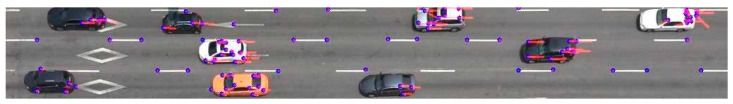
A sample image with motion vectors from an optical flow methodology.

An optical flow method was chosen as the third reference model. Figure 17 shows a sample image with motion vectors after applying an optical flow method based on a 0.1 s time gap. The OpenCV library provides functions relevant to deriving motion vectors from video images. The space mean speed was computed from the length of the motion vectors.

**Figure 18:**
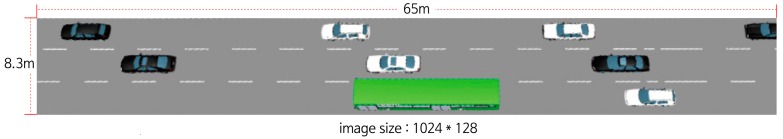
A sample image drawn from the car model provided by Vissim.

Last, a CNN trained on images synthesized by using a 3-D car model was compared with the proposed model. Whereas most traffic simulators provide a simple animation image with cars drawn by a geometric shape, Vissim provides a 3-D car model that draws cars more realistically. Figure 18 shows a sample image drawn by the 3-D car model provided by Vissim.

**Figure 19:**
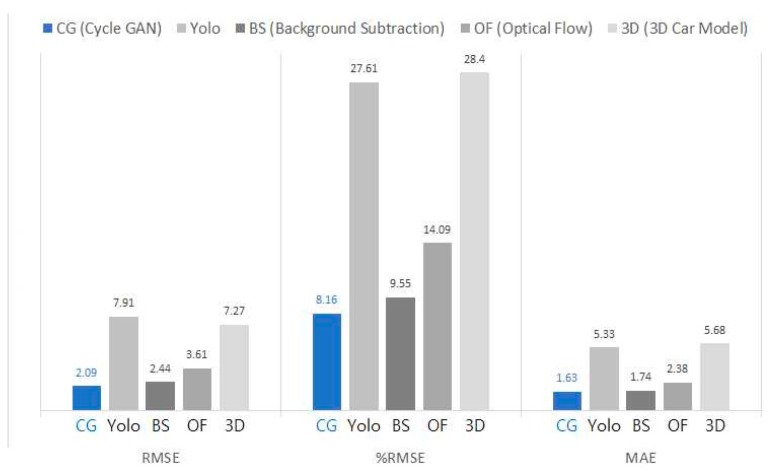
Performance comparisons with other computer vision methodologies.

Figure 19 compares the measurement performances of five different methodologies. Unexpectedly, the model performance from the 3-D car model is the worst. This might be because a car drawn from the 3-D model is more realistic than a dot shapes but is still insufficient to catch up with a true car in real photos. Of course, if a more realistic car model is available, a more accurate result could be obtained. However, it would cost much more to obtain a more realistic 3-D car model than to apply a CycleGAN to synthesizing images.

A YOLO model recorded the second worst performance because it failed to detect crowded vehicles. This does not mean that a YOLO is an unacceptable model for every case. YOLO was pre-trained to detect various kinds of objects in a general photo in a very short time rather than collectively detecting objects in an aerial photo. Using additional bird’s-eye-view images of vehicles to fine-tune the pre-trained YOLO might result in a better performance. However, that would require extra work to tag labels including bounding boxes.

An optical flow method had the third worst performance. The possible reason for the bad performance could be due to the difference in motion vectors for a specific vehicle. The background subtraction method showed good performance in measuring the space mean speed among reference methodologies, although it was inferior to the present approach. Background subtraction is a computer vision technology method that depends on many engineering judgements. The result was based on manual blob recognition. If some rules are applied to identify blobs in an image, the performance of background subtraction methods could be deteriorated. In conclusion, what the comparison results imply is that the proposed approach is fully competitive against other state-of-the-art technologies. It should be noted that the proposed approach requires neither an arbitrary engineering judgement nor a burdensome labeling effort.

## 8. Conclusions

The present study succeeded in measuring the space mean speed using only video images. To the best of our knowledge, there is no other surveillance system that can measure the space mean speed in such a way. The proposed method proved to be the best application of state-of-the-art artificial intelligence. In fact, it will be a long time before artificial intelligence is embedded into applications that have vital influence on human lives such as providing vision for autonomous vehicles or making medical diagnoses. These vital applications demand that artificial intelligence be error-free. However, measuring parameters for traffic operation and management does not require such high-end accuracy. The error-tolerance makes it possible for artificial intelligence at the current levels of technology to be directly used in the field of traffic surveillance. The proposed method of measuring the space mean speed could be the first step in the adoption of incumbent artificial intelligence in solving real-world problems.

Some researchers have assumed that every vehicle running on the road will soon be equipped with an on-board unit that reports their locational information in real time. When this happens, the proposed method might be useless. However, such a connected environment might not soon be realized. Meanwhile, the proposed artificial intelligence could provide traffic parameters in real-time for every road segment where a video camera is available.

The present study dealt only with daily traffic under normal weather conditions. Some challenging conditions such as nighttime, rain, snow, or low lighting will be included in further studies. Among them, we already tested night photos to measure traffic density and the space mean speed. The results are promising and are being prepared for further publications.

In addition, a key to enhancing the performance of the CNN_2_ trained on synthesized images would be to develop a more robust CycleGAN that could generate images that were as close as possible to real photos. However, the performance of CycleGAN fluctuated according to both the model architecture and the hyperparameters used to optimize the loss function. Finding the optimal settings is a labor-intensive task, but the benefits justify the investment.

## Figures and Tables

**Table 1:** The test results of two CNNs trained using synthetic images.

	Root Mean Square Error (RMSE)	Percent Root Mean Square Error (%RMSE)	Map Absolute Error (MAE)
CNN_1_ trained on animation images	1.106	3.858	0.533
CNN_2_ trained on synthesized images	1.473	5.088	0.600

**Table 2:** The test performance of two CNNs when applied to real-world photos.

	Root Mean Square Error (RMSE)	Percent Root Mean Square Error (%RMSE)	Map Absolute Error (MAE)
CNN_1_ trained on animation images	6.306	24.625	5.047
CNN_2_ trained on synthesized images	2.090	8.163	1.630
